# Supplementary material for: Ground-state oxygen holes and the metal–insulator transition in the negative charge-transfer rare-earth nickelates
Source: Nat Commun. 2016 Oct 11;7:13017. doi: 10.1038/ncomms13017 (PMC5062575; doi:10.1038/ncomms13017)
Supplement: Supplementary Information — Supplementary Figures 1-2, Supplementary Table 1, Supplementary Notes 1-4 and Supplementary References [file ncomms13017-s1.pdf]

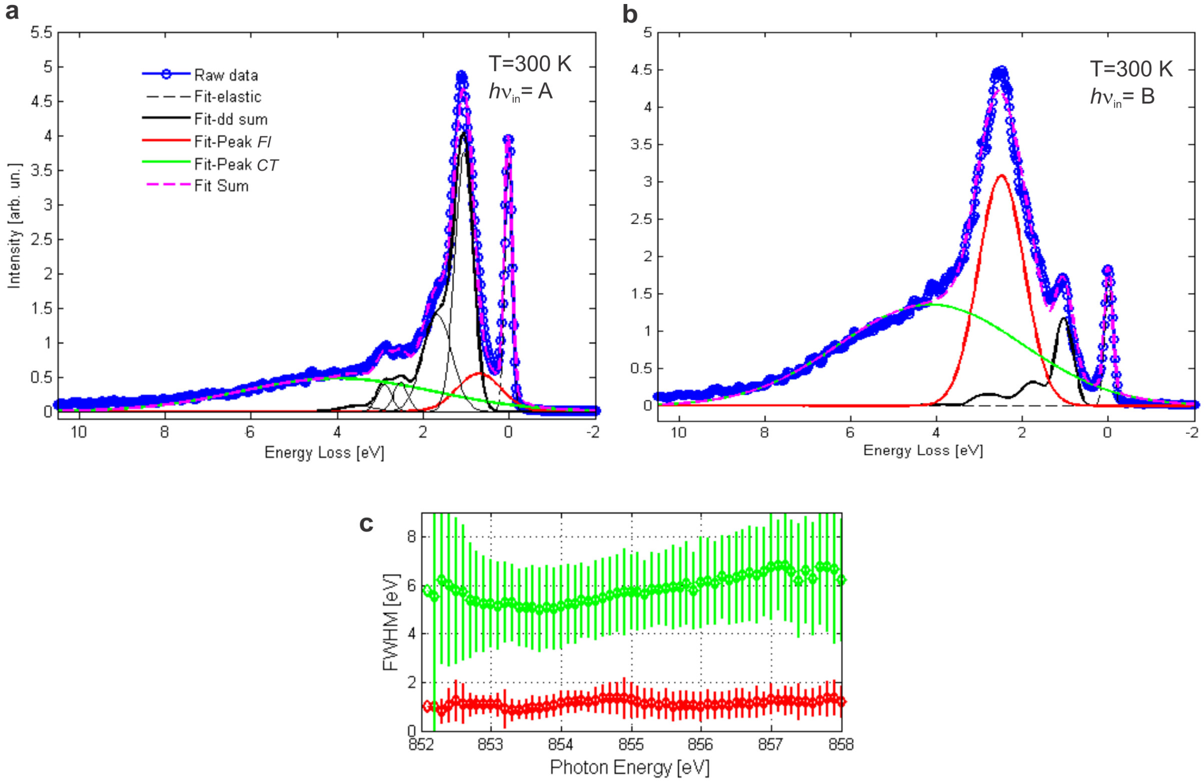


**Supplementary Figure 1 | Details of fitting analysis for RIXS data at 300 K. a**, RIXS spectrum measured at $h\nu_{in}$=A. The thin solid lines refer to the Gaussians used to fit the individual contributions of the spectrum: the elastic line (black dashed line), the intra-band *dd*-excitations (thin black solid lines), a delocalized contribution with a FWHM of 1eV (Peak *Fl* in red) and a charge-transfer contribution with a FWHM of 6 eV (Peak *CT* in green). The black thick solid line represents the sum of the *dd*-contributions. The magenta dashed line represents the sum of all the Gaussian contributions. **b**, RIXS spectrum and similar decomposition as in (a) for $h\nu_{in}$=B. **c**, FWHM of Fl (red) and CT (green) peaks resulting from the fitting analysis, as a function of the Photon Energy at 300 K. The error bars of the FWHM parameters are evaluated using the least square fitting routine and expressed in (s.e.d.).

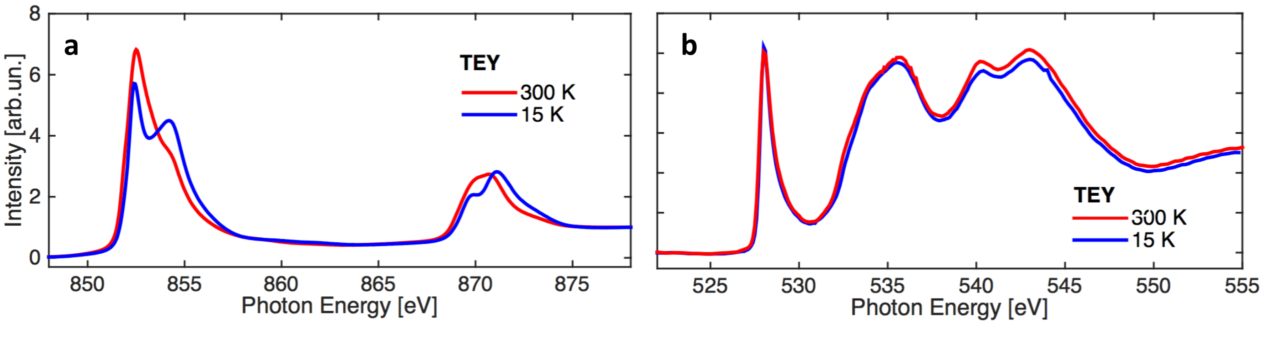


**Supplementary Figure 2 | X-ray Absorption. a,** Ni $L_{3,2}$ X-ray Absorption of $NdNiO_3$ and, **b**, O K XAS in Total Electron Yield. XAS spectrum at 300K (15K) shown in red (blue).

| Parameter | Value | Description |
| --- | --- | --- |
| $F_{dd}^{(2)}$ / $F_{dd}^{(4)}$ | 9.787 / 6.078 | Intra-atomic Slater (Coulomb) integrals of Ni *3d* electrons |
| $F_{2p3d}^{(2)}$ | 7.392 | Intra-atomic Slater (Coulomb) integrals of Ni *2p* and *3d* electrons |
| $G_{2p3d}^{(1)}$ / $G_{2p3d}^{(3)}$ | 5.498 / 3.126 | Intra-atomic Slater (exchange) integrals of Ni *2p* and *3d* electrons |
| $\zeta_{2p}$ / $\zeta_{3d}$ | 11.507 / 0.083 | Spin orbit coupling of the indicated shell |
| $10Dq$ | 0.65 | Ionic crystal field splitting |
| $V_{e_g}$ | 2.06 | Hopping integral for $e_g$ symmetry |
| $V_{t_{2g}}$ | 1.21 | Hopping integral for $t_{2g}$ symmetry |
| $\Delta$ | 4.20 | Charge transfer energy (to center of band) |
| $U_{dd}$ | 6.50 | Monopole part of on-site Coulomb repulsion |
| $U_{pd}$ | 7.50 | Monopole part of *2p* core hole potential |
| $H_{ex}$ | 0.10 | Local exchange field |
| W | 5.40 | Width of ligand valence band |
| N | 25 points | Number of discretization points for ligand band |

**Supplementary Table 1 | Parameters used for the impurity model calculations.** Values are in units of eV, unless otherwise indicated.

## Supplementary Note 1:

## RIXS Spectra fitting analysis

The rich structures of the RIXS spectral lineshapes, represented in Figure 3 (main text) at 15 K and in Supplementary Figure 1 at 300 K (for complementarity), have been analysed and fit by means of spectral decomposition based on Gaussian curves [1]. We use for this analysis the full dataset of the RIXS energy maps. The main prominent RIXS contribution at the XAS resonance $h\nu_{in}$=A (852.4 eV) stems from the localised *dd*-excitations in the range between 1-3 eV. Examining all the RIXS spectra corresponding to incoming photon energies between 852 eV to 859 eV (across the full Ni $L_3$-edge), we notice that the *dd*-excitations do not significantly change in their total profile, but mainly in their intensity. Therefore we determine the *dd*-profile by referring to the resonance spectrum at 852.4 eV [see Suppl. Figure 1 (a)]. Four Gaussian curves (thin black lines) have been tuned to match the sharp peaks emerging from the spectrum in the 1-3 eV energy loss range. Their sum leads to the total *dd*-profile (thick black line). At the same time, the broad residual spectral weights remaining at around 0.7 eV and 4 eV have been fit

with two other Gaussian curves (red and green lines), shallower in intensity but much broader than the previous ones.

Taking advantage of the constant *dd*-profile throughout the Ni $L_3$-edge, we proceed to decompose all the RIXS spectra between 852 eV to 859 eV as the sum of three Gaussians - one for the elastic line (black dashed line), one for *Fl*-peak (red solid line) and one for *CT*- peak (green solid line) – and a properly rescaled *dd*-profile (black thick line) [refer to Suppl. Figure 1 (a-b) for 300K and to Figure 3 (a) from main text for 15K]. The decomposition is done by using a fitting analysis based on the least squares method, and the components listed above define the number of free parameters required for this fitting: (1) the intensity rescaling factor for the *dd*-profile (normalized to unit area); (2-7) the intensity, the peak position and the FWHM of the two Gaussians used respectively to track the *CT* and *Fl* peaks; (8) the intensity of the Gaussian used to fit the elastic peak (the position is by definition at 0eV and its width mostly determined by the experimental resolution).

The extracted fitting parameters as a function of the incident photon energy, and their respective errors, are presented in Figure 3 (main text) and therein discussed in terms of intensity [Figure 3 (b-c)] and peak position [Figure 3(d-e)]. In addition, we plot in Suppl. Figure 1 (c) the extracted FWHM for the *CT* and the *Fl* peaks. We note that these values remain reasonably constant (±10%) in the full energy range and average at 1 eV for *Fl*- and 6 eV for the *CT*-peak. Such a result further supports the validity of the presented decomposition analysis, allowing characterizing the energy dispersion of the *CT* and *Fl* contributions as a function of the incident photon energy.

**Supplementary Note 2:**

**X-ray Absorption in Total Electron Yield**

X-ray absorption measurements in Total Electron Yield mode (TEY) have been performed for $NdNiO_3$. Spectra acquired for 300K and 15K are presented in Suppl. Figure 2, both at Ni L and O K edges. Given the strong surface-sensitivity of the TEY signal, we used in the main part of the

paper the Partial Fluorescence Yield (PFY) obtained by integrating the RIXS spectra at the Ni $L_3$-edge, to ensure the bulk-sensitivity. The XAS in PFY mode displayed in Figure 2(a) of the main text is in good agreement with previous data published in the literature [2, 3]. This confirms the good quality of our sample. Concerning the O K XAS spectra, our measurements displayed in Supplementary Figure 2(b) are also in good agreement with the main features observed in the literature, on the bulk $NdNiO_3$ sample [2, 3], thus showing: 1) a very strong pre-peak around 528 eV which is proportional to the covalent degree in the system; 2) a small spectral weight increase at the prepeak and a decrease at the 536 eV peak, while going to the insulating phase [3].

**Supplementary Note 3:**

**Cluster and Crystal Filed calculations for orbital excitations in $NdNiO_3$**

The spectral shape of the *dd* excitations is strongly determined by the local Ni 3*d* orbital energies and occupations, and accordingly provide direct insight into the Ni valence. To analyze the observed *dd* excitations, we first computed the RIXS response of a typical $3d^7$ cluster model, which has been used to interpret the XAS of the nickelates in the past [3]. In this model, a $3d^7$ Ni impurity hybridizes with a fully occupied oxygen octahedron, leading to a mixed, many-body ground state wavefunction of the form $|\psi_0\rangle = \alpha|d^7\underline{L}^0\rangle + \beta|d^8\underline{L}^1\rangle + \gamma|d^9\underline{L}^2\rangle$, where $\underline{L}$ denotes a ligand hole. We took all model parameters (multipole Coulomb interactions, crystal field splitting, hopping integrals, charge-transfer energies) from Ref. [3]. RIXS spectra were calculated using the Kramers-Heisenberg equation. The spectra were broadened with Lorentzian and Gaussian lineshapes to account for lifetime and instrumental broadening effects, respectively. We find that the computed RIXS spectra show *dd* excitations which strongly disagree with experiment, including a low energy low-spin *to* high-spin excitation near 0.25 eV energy loss which is not found in experiment, and other multiplets which deviate strongly from those observed experimentally.

Next, we calculated the expected *dd* excitations from a Ni $3d^8$ configuration having the local $O_h$ symmetry (approximately) present in the nickelates. We find that even using a simple crystal field model, excellent agreement with the experimental *dd* excitations is obtained, by employing model parameters (multipole Coulomb interactions, crystal field splitting) which have been used in studies of NiO [4]. In particular, we have for the Slater integrals $F^2$ = 8.063 eV and $F^4$ = 5.699 eV, and for the crystal field splitting a value of $10D_q$ = 1.05 eV. Including hybridization effects would add charge-transfer excitations, but would not strongly alter the *dd* excitations after a renormalization of the crystal field energy [4].

**Supplementary Note 4:**

**Singe Impurity Anderson Model calculations**

The Single Impurity Anderson Model (SIAM) calculations used (in Figure 6 and related discussion in the main manuscript) to analyze the behaviour of local *dd* and nonlocal charge-transfer excitations include full Ni 3*d*-shell multiplets and coupling to a fully occupied, ligand O 2*p*-like valence band. The model parameters used are shown in Supplementary Table 1 and are typical for a NiO-type system, agreeing well with both *ab-initio* NiO values [5] and those fitted to experiment [6,7]. Note, however, that the precise values for the parameters are not important for the analysis presented in this work. For the $3d^8$ impurity configuration considered here, the basis sizes are relatively small (of order $10^3$), thus easily allowing full diagonalization of the Hamiltonian matrices for each irreducible representation. XAS and RIXS spectra were then calculated using Fermi's Golden Rule #2 and the Kramers-Heisenberg equation, respectively [8]. The spectra were broadened with Lorentzian and Gaussian lineshapes to account for lifetime and instrumental broadening effects, respectively.

**Supplementary References:**

**1.** Lee, J. J. et *al*. Charge-orbital-lattice coupling effects in the *dd* excitation profile of one-dimensional cuprates. *Phys. Rev. B* **89**, 041104 (R) (2014).

**2.** Medarde, M. et *al.* $RNiO_3$ perovskites (R =Pr,Nd): Nickel valence and the metal-insulator transition investigated by x-ray-absorption spectroscopy. *Phys. Rev. B* **46**, 14975 (1992)

**3.** Freeland, J. W., van Veenendaal, M. and Chakhalian, J. Evolution of electronic structure across the rare-earth $RNiO_3$ series. *J. Electron. Spectrosc. Relat. Phenom*. 208, 56 (2016).
**4.** Ghiringhelli, G. et *al*. NiO as a test case for high resolution resonant inelastic soft x-ray scattering. *J. Phys.: Condens. Matter* **17**, 5397 (2005).
**5.** Haverkort, M. W., Zwierzycki, M. W., and Andersen, O. K. Multiplet ligand-field theory using Wannier orbitals. *Phys. Rev. B* **85**, 165113 (2012).
**6.** Matsubara, M., Uozumi, T., Kotani, A. and Parlebas, J. C. Charge Transfer Excitation in Resonant X-ray Emission Spectroscopy of NiO. *J. Phys. Soc. Japan* **74**(7), 2052 (2005).
**7.** Das, S. C. et *al.* Band Gap Tuning in ZnO Through Ni Doping via Spray Pyrolysis. *J. Phys. Chem. C* **117**, 12745, (2013).
**8.** Ament, L. J. P., van Veendendaal, M., Devereaux, T. P., Hill, J. P. and van den Brink, J. Resonant inelastic x-ray scattering studies of elementary excitations. *Rev. Mod. Phys.* **83**, 705 (2011).
